# Osteoporosis in a 60-Year-Old Male With a History of Chronic Myeloid Leukemia Treated With Imatinib Mesylate

**DOI:** 10.7759/cureus.40368

**Published:** 2023-06-13

**Authors:** Jaskaran Batra, Anvitha R Ankireddypalli, Ashok Kumar Kanugula, Swathi Gorle, Jasleen Kaur

**Affiliations:** 1 Internal Medicine, University of Pittsburgh Medical Center (UMPC) McKeesport, McKeesport, USA; 2 Endocrinology, University of Minnesota School of Medicine, Minneapolis, USA; 3 Internal Medicine, Wellstar Spalding Regional Medical Center, Griffin, USA; 4 Endocrinology, HealthPartners, Minneapolis, USA

**Keywords:** mineral bone metabolism, chronic myelogenous leukaemia (cml), verterbal compression fracture, imatinib mesylate, osteoporosis

## Abstract

Secondary osteoporosis is defined as a decline in bone mineral density due to any underlying etiology, which usually results in accelerated bone loss than expected for the individual’s age or gender. Almost 50-80% of men diagnosed with osteoporosis have secondary osteoporosis. We present a case of a 60-year-old male with secondary osteoporosis with a history of imatinib mesylate-treated chronic myeloid leukemia (CML). Imatinib mesylate has revolutionized the management of individuals with chronic myeloid leukemia, which is now managed as a chronic disease. Imatinib has been demonstrated to cause dysregulation of bone metabolism. The long-term effects of imatinib on bone metabolism are still unknown.

## Introduction

Primary osteoporosis is due to the normal age-related decline of bone mineral density, also associated with postmenopausal and age-related decline in sex hormones in women and men, respectively. Secondary osteoporosis is defined as a decline in bone mineral density due to any underlying etiology, which usually results in accelerated bone loss than expected for the individual’s age or gender [1]. Approximately 30% of postmenopausal women, half of premenopausal women, and 50-80% of men diagnosed with osteoporosis have secondary osteoporosis [2]. If the underlying cause remains unidentified, osteoporosis treatment in these individuals is ineffective. Hence, identifying and treating these causes are necessary, if possible. Postmenopausal women and men above the age of 50 years are diagnosed with osteoporosis when the T score is ≤2.5 at the hip, lumbar spine, or forearm. In addition, individuals with secondary osteoporosis can be identified when they have a Z score ≤2.0, which represents significantly lower bone density than age- and gender-matched controls and should have an evaluation for underlying causes of osteoporosis [3]. Early identification and appropriate management of osteoporosis are essential due to the increased morbidity and mortality associated with osteoporotic fractures [4, 5].

Chronic myeloid leukemia (CML) is a common myeloproliferative neoplasm, with 85% of the patients presenting in the chronic phase. This leukemia is associated with the BCR-ABL fusion gene [6]. The use of ABL1 kinase inhibitors generates a dramatic response in patients presenting in this phase, significantly improving outcomes and survival rates [6]. We present a case of a 60-year-old male with secondary osteoporosis with a history of imatinib mesylate-treated chronic myeloid leukemia (CML).

## Case presentation

We present the case of a 60-year-old male who had presented for evaluation of osteoporosis. This was diagnosed after he was found to have an L1 burst fracture during evaluation for back pain. The patient was a small airplane co-pilot involved in a forced landing when its engine seized about 150 feet off the ground. He developed acute pain in his back upon this impact. His medical history was significant for chronic myeloid leukemia, diagnosed in 2015, and benign prostatic hyperplasia. His medications include aspirin 81 mg daily, multivitamins, and imatinib 400 mg daily (starting in July 2015). No history of chronic systemic steroid therapy. He did not have a history of bone marrow transplants for CML. He does not smoke or drink alcohol. He did not report any symptoms suggestive of hypogonadism. He had an adequate calcium intake through diet via various dairy products, green leafy vegetables, and whole nuts. No known family history of osteoporosis. His mother passed away at 53 years of age, and his father at 68 years of age, both from a diagnosis of lung carcinoma.

**Figure 1:**
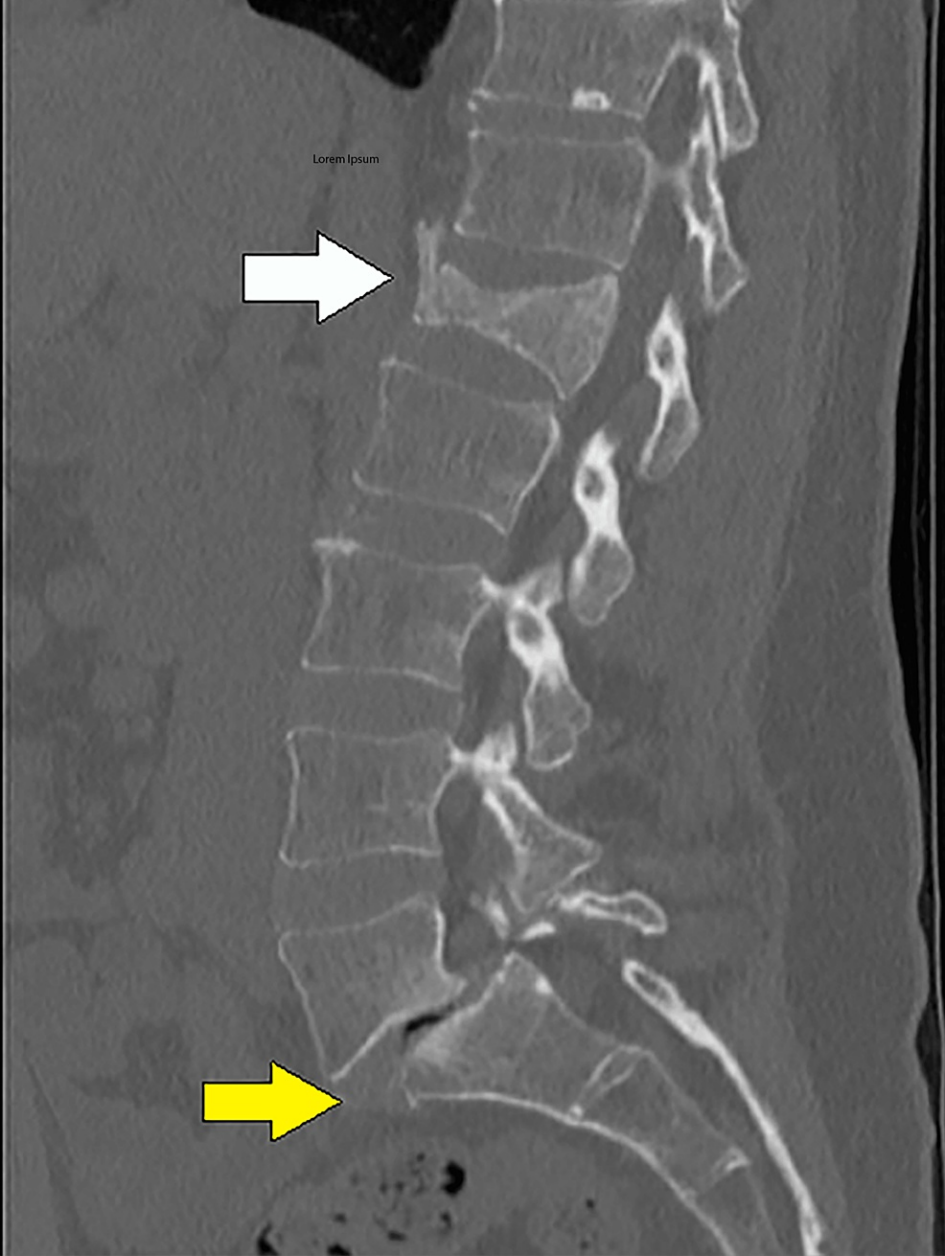
CT scan of the lumbar spine White arrow: L1 compression fracture; Yellow arrow: L5 pars defect

**Figure 2:**
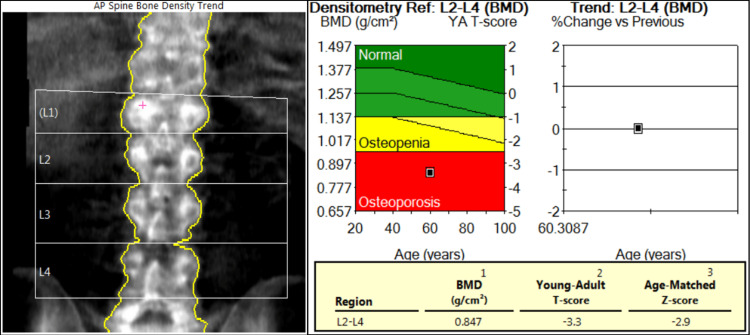
DXA results for lumbar spine BMD: bone mineral density

**Figure 3:**
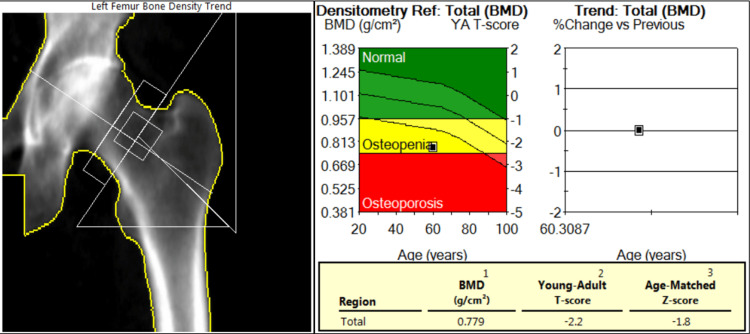
DXA scan of the left hip BMD: bone mineral density

Computed tomography (CT) of the lumbar spine revealed an acute two-column burst fracture of the L1 vertebral body with up to 65% loss of vertebral body height centrally and minimal posterior retropulsion (Figure 1). No extension of the fracture to involve the posterior elements. Bilateral chronic L5 pars defects with 11 mm anterolisthesis of L5 on S1. Diffuse osteopenia was also noted. After two months, an 80% height loss of the L1 vertebra was demonstrated centrally with slight interval worsening of mild retropulsion on repeat CT of the lumbar spine. Dual X-ray absorptiometry (DXA) showed a T score of -3.3 and a Z score of -2.9 at the lumbar spine (L2-L4), L1 was excluded due to compression fracture (Figure 2). At the left total hip, the T score was -2.2, and the Z score was -1.8; at the left femur neck, the T score was -2.3, and the Z score was -1.3 (Figure 3). No previous DXA scans were performed. Bone density at the forearm was not evaluated.

An extensive laboratory evaluation was performed to evaluate for any secondary causes of osteoporosis, given the patient’s age, gender, and Z score <2.0 at the lumbar spine on the DXA scan (Tables 1, 2). He had a decline in serum phosphate levels compared to baseline (before initiation of imatinib, see Table 1). Serum calcium levels were approximately similar at baseline and after seven years (Table 1). We did not evaluate parathyroid hormone levels at baseline. After seven years of imatinib therapy, parathyroid hormone levels were normal.

**Table 1:** Comparison of bone labs before and after initiation of osteoporosis GFR: glomerular filtration rate

Test	Pre-fracture values (in 2015)	Post-fracture values (in 2022)	Normal Range
Calcium	9.2	8.9	8.4-10.4 mg/dL
Phosphorus	3.6	2.3	2.3-4.7 mg/dL
Creatinine	0.80	0.81	0.73-1.18 mg/dL
Estimated GFR	>60	>60	>60 mL/min/1.73m2
25-hydroxy Vitamin D3	50.8	62	30-80 ng/mL

**Table 2:** Laboratory evaluation for secondary causes of osteoporosis TSH: thyroid stimulating hormone; HbA1c: hemoglobin A1c; AST: asparate aminotransaminase; ALT: alanine aminotransaminase

Test	Value (in 2022)	Normal Range
Hemoglobin	14.2	13.5-17.5 g/dL
Red blood cell count	4.40	4.32-5.72 million cells/µL
White blood cell count	4600	3500-10500 cells/µL
Platelet count	229,000	150,000-450,000 cells/µL
Magnesium	1.8	1.6-2.6 mg/dL
Parathyroid hormone	71	10-100 pg/mL
TSH	0.93	0.3-4.50 mIU/mL
Alkaline Phosphatase, Total	58	40-150 U/L
Testosterone, total	245	200-745 ng/dL
Testosterone, free	3.5	3.1-12.8 ng/dL
Testosterone, bioavailable	98.4	71.7-300.0 ng/dL
Sex hormone binding globulin	36	13-74 nmol/L
Glucose, Random	116	<140 mg/dL
HbA1c	5.5	<=5.6%
Bilirubin, Total	0.7	0.2-1.2 mg/dL
AST	24	10-40 U/L
ALT	23	0-55 U/L
Total protein	7.2	6.4-8.3 g/dL
Albumin	4.6	3.5-5.0 g/dL
Serum protein electrophoresis	No monoclonal protein detected	
Kappa free light chains	1.09	0.33-1.94 mg/dL
Lambda-free light chains	0.82	0.57-2.63 mg/dL
Kappa/Lambda ratio	1.33	0.26-1.65
Urine calcium, 24 hours	26.0	<=60.0 µg/24 hr
Urine cortisol, 24 hours	290	100-300 mg/24 hr
Tissue Transglutaminase Antibody, IgA	<1.0	0.0-6.9 U/mL
IgA, serum	232	65-421 mg/dL
BCR/ABL1 Major P210	Detected	
BCR/ABL1 International Scale %	0.0086	

The patient was recommended treatment with Romosozumab for the management of osteoporosis. In addition, Imatinib therapy was continued for the management of CML.

## Discussion

Imatinib mesylate and other ABL1 kinase inhibitors have revolutionized the management of individuals with chronic-phase CML [7]. This disease is now managed as a chronic disease, and patients can expect a near-normal life expectancy [8]. As a result, there has been an increase in the prevalence of CML as patients with CML are living longer [6]. The prevalence of CML is expected to rise to approximately 180,000 cases by 2030-2040 [9]. Imatinib has been demonstrated to cause dysregulation of bone metabolism.

In effect of Imatinib in patients with CML is due to its ability to inhibit the activity of tyrosine kinase BCR-ABL2, a fusion oncoprotein resulting from the translocation between chromosomes 9 and 22. It is also a competitive inhibitor of other tyrosine kinases: c-kit, M-CSF (macrophage-colony stimulating factor), PDGFα (platelet-derived growth factor), and PDGFβ [10]. M-CSF, PDGFα, and PDGFβ are involved in the differentiation and activity of osteoblasts and osteoclasts [11]. M-CSF is involved in the early proliferative phase of osteoclastogenesis by affecting the action of receptor activators of nuclear factor κB ligand (RANKL) [11]. PDGFα is expressed by mesenchymal stromal cells, which have the potential to differentiate into osteoblasts. PDGFβ is expressed by vascular smooth muscle cells and pericytes and is involved in angiogenesis in the bone [11]. Evidence from in vivo and in vitro experiments demonstrates decreased osteoclast number and activity due to imatinib leading to reduced bone resorption [12]. However, the effect on osteoblasts is less clear. There have been mixed results from in vitro and in vivo experiments [12]. Short-term studies have shown calcium and phosphate metabolism alteration using imatinib [13]. Imatinib-treated patients can have lower serum calcium levels, which can cause compensatory secondary hyperparathyroidism contributing to bone resorption [12]. Imatinib use can also cause hypophosphatemia, possibly due to secondary hyperparathyroidism and decreased renal phosphate reabsorption [14].

Berman et al. had prospectively followed nineteen patients treated with imatinib mesylate to manage CML or gastrointestinal stromal tumors for two years [15]. Thirteen patients were on a dose of 400 mg/day, five on a dose higher than 400 mg/day, and the remaining on 100 mg/day. They checked metabolic markers of bone synthesis, resorption, and serial bone mineral density (BMD) by DXA scans. Nine out of nineteen patients had a decline in BMD, four patients had an increase in BMD, and the remaining six had stable BMD. These changes were independent of age, osteocalcin levels, levels of serum N-telopeptide of type 1 collagen cross-links (NTX), and phosphorus levels. In addition, seven of the nine patients who had a decline in BMD had a significant decrease at the total hip, and four had a decline at the femur neck. No significant change was seen in the lumbar spine. However, this study did not evaluate the mechanisms possibly responsible for changes in BMD.

A substudy of the Therapeutic Intensification in de Novo Leukemia (TIDEL) II trial assessed the effect of high dose imatinib mesylate, 600 mg daily, on BMD evaluated by serial DXA scans in eleven patients treated for CML at 6, 12, and 24 months [16]. All patients had low phosphorus levels and a decline in serum calcium levels compared to baseline. All patients also had an elevation in parathyroid hormone levels. All patients had stable BMD at 24 months at the lumbar spine, total hip, and total body compared to baseline. However, nine of the eleven patients had an increase in BMD at the distal 33% radius. Ten out of eleven patients have a significant decline in BMD at the femur neck. This study found a significant decrease in osteoclast number and activity based on bone biopsy results and levels of serum of C-terminal collagen crosslinks (CTX). There was no effect on osteoblast activity based on bone biopsy and bone alkaline phosphatase and procollagen 1 intact N-terminal propeptide (P1NP) levels. Yet, these biochemical markers had a downward trend at 24 months. Another 18-month prospective study involving nine patients treated with 400 mg imatinib for CML studied its effects on bone markers and BMD [14]. Serum calcium and phosphate levels were significantly lower than the baseline, and PTH levels were significantly higher at 3, 6, and 18 months. The bone formation markers, osteocalcin, and P1NP levels were significantly higher in the initial months and became similar to baseline at 18 months. The bone resorption marker, CTX, was similar at 3 and 6 months but significantly lower than baseline at 18 months. BMD (measured by DXA) increased at the lumbar spine at 18 months compared to baseline but remained unchanged at the proximal femur and total body.

These studies have been performed on a very small group of patients for short durations (approximately two years). This can be explained by the low incidence of CML, 9000 cases/year [8]. There have yet to be any recent large-scale studies evaluating the long-term effects of imatinib on metabolic bone markers and BMD. Our patient was treated with imatinib for seven years to manage CML before being diagnosed with osteoporosis.

## Conclusions

Imatinib mesylate is being used as chronic therapy in patients with CML. It is known to cause dysregulation of bone metabolism. Most studies evaluating this medication’s effects on bone metabolism and bone mineral density (assessed by DXA scan) have been short-term and have included a very small number of patients. As more patients with CML are on imatinib for longer periods of time, they can be predisposed to the long-term effects of imatinib on bone metabolism. These long-term effects need to be evaluated in the future to determine if these patients need a periodic evaluation of their BMD by serial DXA scans.

## References

[1] Fitzpatrick LA (2002). Secondary causes of osteoporosis. Mayo Clin Proc.

[2] Hudec SM, Camacho PM (2013). Secondary causes of osteoporosis. Endocr Pract.

[3] Ebeling PR, Nguyen HH, Aleksova J, Vincent AJ, Wong P, Milat F (2022). Secondary osteoporosis. Endocr Rev.

[4] Abrahamsen B, Osmond C, Cooper C (2015). Life expectancy in patients treated for osteoporosis: observational cohort study using national Danish prescription data. J Bone Miner Res.

[5] Bliuc D, Nguyen ND, Milch VE, Nguyen TV, Eisman JA, Center JR (2009). Mortality risk associated with low-trauma osteoporotic fracture and subsequent fracture in men and women. JAMA.

[6] Jabbour E, Kantarjian H (2020). Chronic myeloid leukemia: 2020 update on diagnosis, therapy and monitoring. Am J Hematol.

[7] Kantarjian H, Sawyers C, Hochhaus A (2002). Hematologic and cytogenetic responses to imatinib mesylate in chronic myelogenous leukemia. N Engl J Med.

[8] Maas CC, van Klaveren D, Ector GI (2022). The evolution of the loss of life expectancy in patients with chronic myeloid leukaemia: a population-based study in the Netherlands, 1989-2018. Br J Haematol.

[9] Huang X, Cortes J, Kantarjian H (2012). Estimations of the increasing prevalence and plateau prevalence of chronic myeloid leukemia in the era of tyrosine kinase inhibitor therapy. Cancer.

[10] Cohen P, Cross D, Jänne PA (2021). Kinase drug discovery 20 years after imatinib: progress and future directions. Nat Rev Drug Discov.

[11] Ikeda K, Takeshita S (2016). The role of osteoclast differentiation and function in skeletal homeostasis. J Biochem.

[12] Alemán JO, Farooki A, Girotra M (2014). Effects of tyrosine kinase inhibition on bone metabolism: untargeted consequences of targeted therapies. Endocr Relat Cancer.

[13] Berman E, Nicolaides M, Maki RG (2006). Altered bone and mineral metabolism in patients receiving imatinib mesylate. N Engl J Med.

[14] O'Sullivan S, Horne A, Wattie D (2009). Decreased bone turnover despite persistent secondary hyperparathyroidism during prolonged treatment with imatinib. J Clin Endocrinol Metab.

[15] Berman E, Girotra M, Cheng C (2013). Effect of long term imatinib on bone in adults with chronic myelogenous leukemia and gastrointestinal stromal tumors. Leuk Res.

[16] Vandyke K, Fitter S, Drew J (2013). Prospective histomorphometric and DXA evaluation of bone remodeling in imatinib-treated CML patients: evidence for site-specific skeletal effects. J Clin Endocrinol Metab.

